# Identification of potential biomarkers of myopia based on machine learning algorithms

**DOI:** 10.1186/s12886-023-03119-5

**Published:** 2023-09-22

**Authors:** Shengnan Zhang, Tao Wang, Huaihua Wang, Bingfang Gao, Chao Sun

**Affiliations:** 1 Department of Ophthalmology, Zibo Central Hospital, No.54, Gongqingtuan West Road, Zhangdian District, Zibo, 255000 Shandong Province PR China; 2 Sanitary Inspection Center, Zibo Center for Disease Control and Prevention, Zibo, 255000 PR China; 3 Department of Pathology, Zibo Hospital of Integrated Traditional Chinese and Western Medicine Zibo, Zibo, 255000 PR China

**Keywords:** Myopia, Machine learning, Biomarkers, Gene expression, Diagnosis

## Abstract

**Purpose:**

This study aims to identify potential myopia biomarkers using machine learning algorithms, enhancing myopia diagnosis and prognosis prediction.

**Methods:**

GSE112155 and GSE15163 datasets from the GEO database were analyzed. We used “limma” for differential expression analysis and “GO plot” and “clusterProfiler” for functional and pathway enrichment analyses. The LASSO and SVM-RFE algorithms were employed to screen myopia-related biomarkers, followed by ROC curve analysis for diagnostic performance evaluation. Single-gene GSEA enrichment analysis was executed using GSEA 4.1.0.

**Results:**

The functional analysis of differentially expressed genes indicated their role in carbohydrate generation and polysaccharide synthesis. We identified 23 differentially expressed genes associated with myopia, four of which were highly effective diagnostic biomarkers. Single gene GSEA results showed these genes control the ubiquitin-mediated protein hydrolysis pathway.

**Conclusion:**

Our study identifies four key myopia biomarkers, providing a foundation for future clinical and experimental validation studies.

**Supplementary Information:**

The online version contains supplementary material available at 10.1186/s12886-023-03119-5.

## Introduction

Myopia, or nearsightedness, is a common vision condition where close objects appear clear, but distant ones are blurred. It increases the risk of several eye-related complications such as retinal detachment, dry eye, cataracts, and glaucoma. Additionally, symptoms like headaches and eye strain can occur [1–3]. With a global prevalence of 34% in 2020, projected to rise to 49.8% by 2050, myopia presents a significant public health challenge worldwide [4].The prevalence of myopia is rising year over year in several populations as a result of changes in people’s lifestyles and daily routines [5]. Myopia is caused by a complex interaction of hereditary and environmental variables, which are now thought to be the main cause.

Several studies have examined the relationship between mutations in disease-causing genes and myopia. A study collected data from 593 individuals with high myopia for gene-set analysis (GSA) of new genome-wide association study (GWAS) data and identified by whole-genome sequencing 45 triplet families with high myopia, screening 196 genes with ab initio mutations for over-representation analysis (ORA), and 284 previously reported myopia risk genes for ORA for human genetic analysis. At last, it implicated the HIF-1α signaling pathway in promoting human myopia through mediating interactions between genetic and environmental factors [6]. The SOX2 gene’s rs4575941 allele G, which may be a risk gene for high myopia in the Chinese population, was predicted to play some roles in the genetic vulnerability to high myopia [7]. PAX6 has recently been identified as a myopia-risk gene by meta-analysis. Additionally, it found a strong link between PAX6 and HOXA9. In addition, it has been noted that HOXA9 activates TGF, a risk factor for myopia. HOXA9 may encourage pro-myopia gene expression and RPE growth, which ultimately aid in the development of myopia [8].

Additional data from a study support the hypothesis that the PAX6 SNP rs644242 is linked to severe myopia. The gene may contribute to the emergence or progression of severe myopia [9]. Loss of VIPR2 function may impair bipolar cell function, which corresponds to an increase in form deprivation myopia (FDM), and thus the VIP-VIPR2 signaling pathway axis is a viable new target to control the development of this condition [10]. Myopia is currently treated both domestically and internationally mostly with corrective surgery, medicine, and frame glasses. Sports medicine and vision care still lack significant experience. Recent developments in biology have led to the identification of numerous loci and mutations or variants linked to myopia using molecular approaches such as linkage analysis, candidate gene identification, GWAS, and next-generation sequencing (NGS) [11].

The increasing prevalence of myopia has accelerated our research on the pathogenesis of myopia. To further investigate the mutated genes in the corneas of myopic samples, we explored the differences in gene expression between myopic and normal corneas to discover the molecular biological mechanism of myopia pathogenesis and precisely target myopia treatment to provide a reference for clinical treatment of myopia.

## Methods

### Data sources

Data from the Gene Expression Omnibus (GEO) (https://www.ncbi.nlm.nih.gov/geo/) database in the GSE112155 and GSE151631 datasets were used for analysis in this study. Gene expression levels were normalized using transcripts per kilobase million (TPM) values; the following equation was used: TPM = Read count × 1,000,000/Mapped Reads [12].

### Analysis methods

#### Analysis of differentially expressed genes

Differential expression analysis was performed on control samples from GSE112155 and Keratoconus patient samples from GSE151631 using the “limma” package, and the genes that were up- and down-regulated in the two datasets were plotted separately in a Venn diagram, with the overlap identified as differentially expressed genes associated with myopia.

#### Functional and pathway enrichment analysis

Gene Ontology (GO) and Kyoto Encyclopedia of Genes and Genomes (KEGG) enrichment analyses were performed for differentially expressed genes using the “GO plot” and “clusterProfiler” software packages. The GO enrichment analysis includes cell composition (CC), biological process (BP), and molecular function (MF).

#### Selecting myopia-related biomarkers

The software packages “glmnet” and “e1071” were used to perform Support Vector Machine Recursive Feature Elimination (SVM-RFE) analysis and Least Absolute Shrinkage and Selection Operator (LASSO) Logistic Regression. Two machine learning algorithms were used to screen the biomarkers, and the genes they identified were then shown in a Venn diagram with the myopia-related biomarkers occupying the overlapped areas. The diagnostic effectiveness of the biomarkers was then tested by plotting the receiver operating characteristic (ROC) and measuring the AUC.

#### Biomarker-related pathway prediction

To predict biomarker-related pathways software GSEA_4.1.0 was used to perform single-gene GSEA enrichment analysis. The GSE112155 and GSE151631 data were first merged and platform effects were eliminated, and the four biomarkers were divided into high and low-expression groups based on their expression, respectively. The significance criterion was a nominal *P*-value < 5%.

## Results

### Analysis of differentially expressed genes

**Fig. 1:**
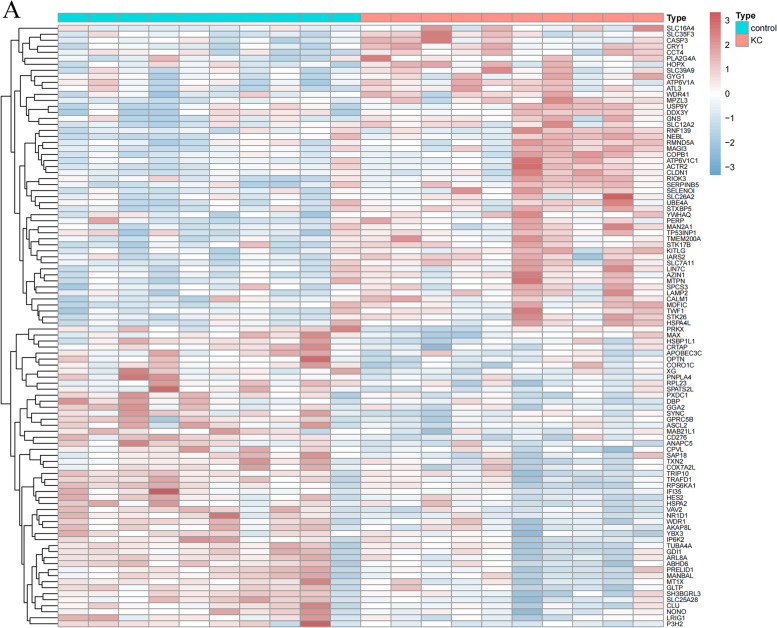

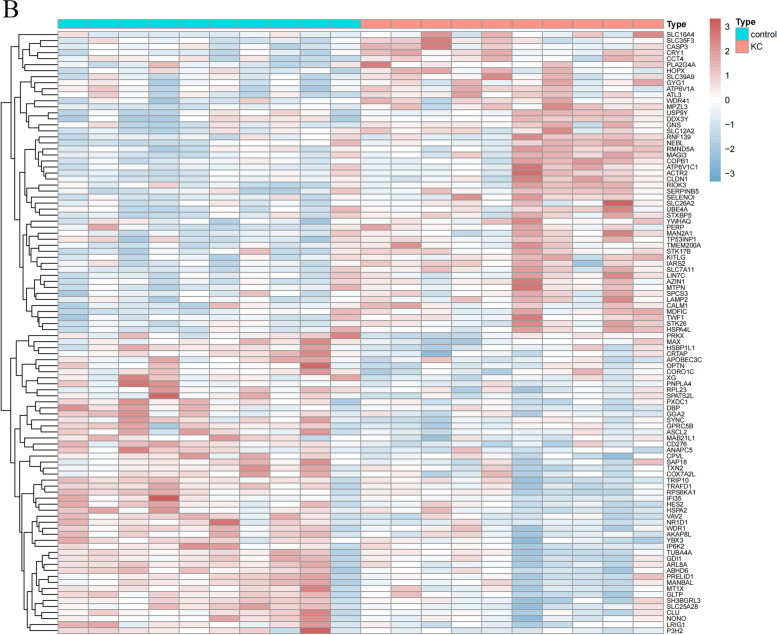
Differential expression analysis. **A** Heatmap of differential expression analysis of GSE112155 dataset. **B** Heatmap of differential expression analysis of GSE151631 dataset

The datasets GSE112155 and GSE151631 were transformed into TPM for differentially expressed gene analysis. 308 differentially expressed genes were identified in GSE112155 (Fig. 1A), which contained 189 up-regulated genes and 119 down-regulated genes; 1848 differentially expressed genes were identified in GSE151631, which contained 699 up-regulated genes and 1149 down-regulated genes (Fig. 1B).

**Fig. 2:**
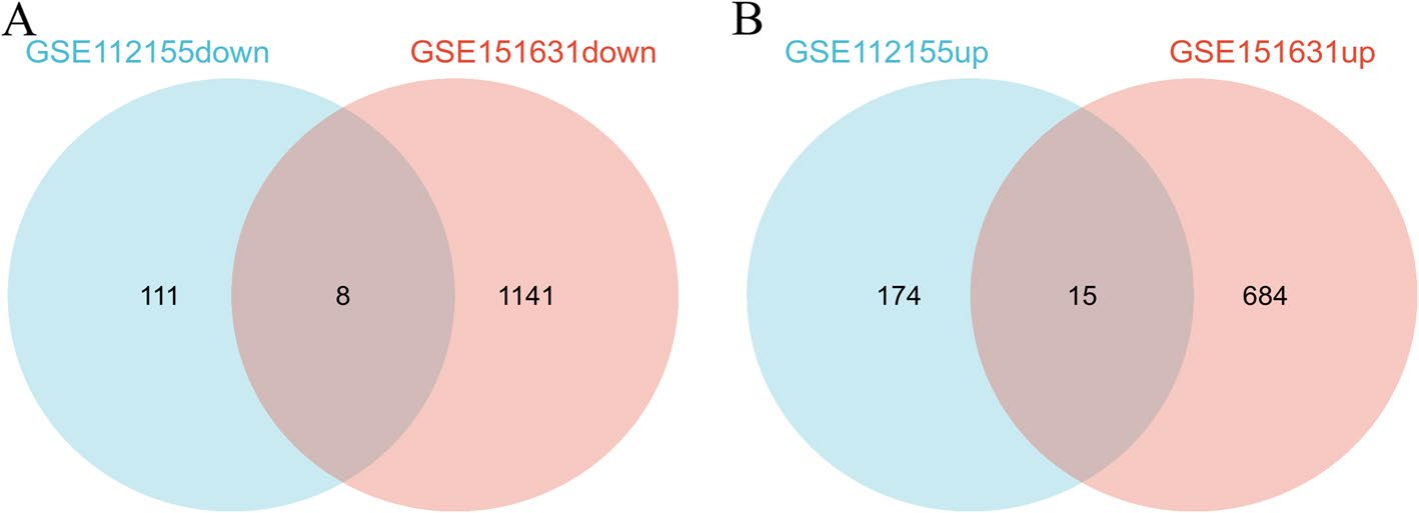
Venn diagram of differentially expressed genes in both datasets. **A** Venn diagram of down-regulated genes in GSE112155 and GSE151631. **B** Venn diagram of up-regulated genes in GSE112155 and GSE151631. **C** Venn diagram of up-regulated genes in GSE112155 and GSE151631

The datasets GSE112155 and GSE151631 up-regulated and down-regulated genes were plotted separately on a Venn diagram to take the intersection, and eight genes that were co-regulated in GSE112155 and GSE151631 (Fig. 2A) and 15 genes that were co-regulated in GSE112155 and GSE151631 (Fig. 2B) were obtained. The latter 23 genes were identified as myopia-related differentially expressed genes.

### Functional and pathway enrichment analysis

**Fig. 3:**
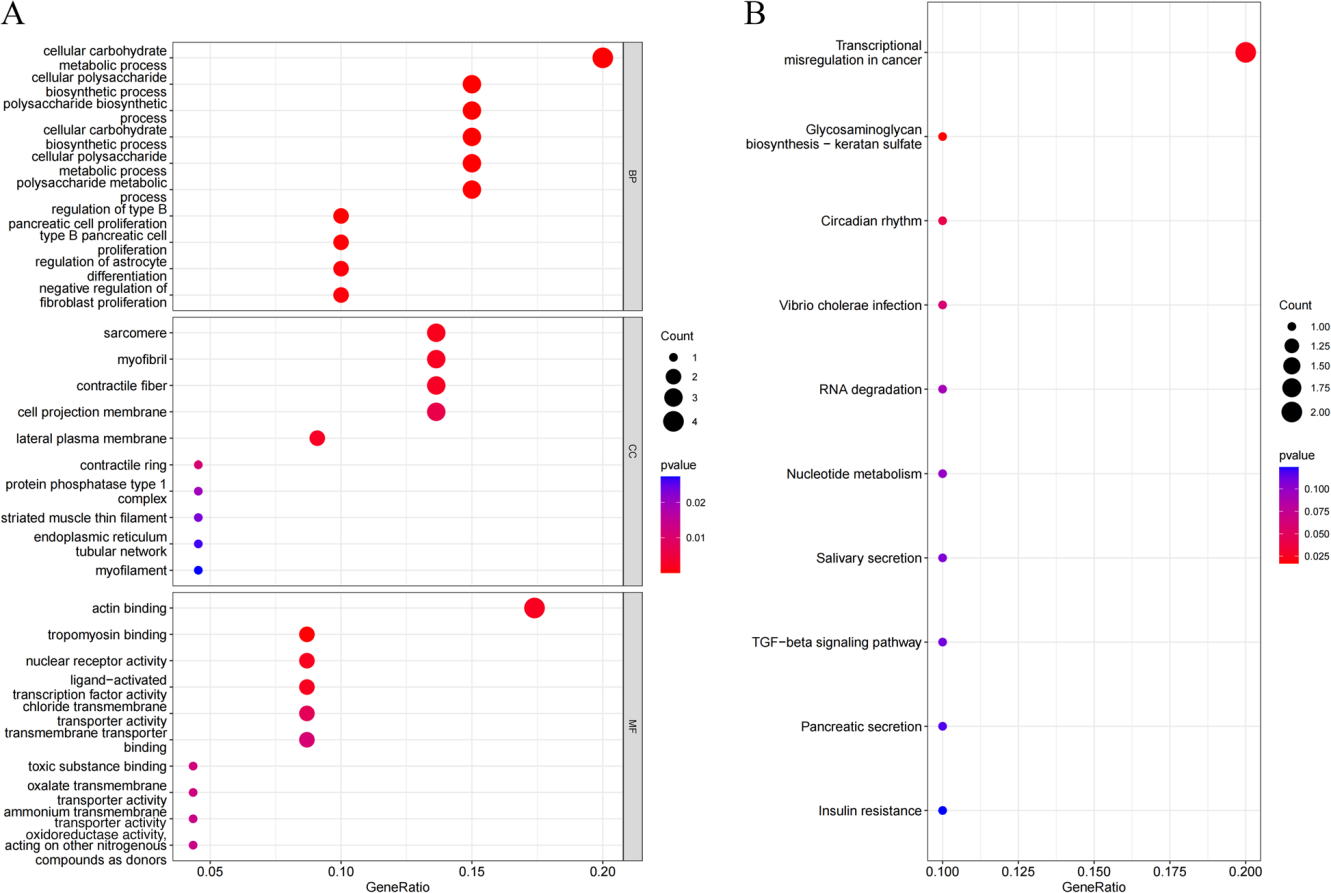
Functional and pathway enrichment analysis of differentially expressed genes. **A** GO enrichment analysis. **B** KEGG enrichment analysis

Then, we conducted functional and pathway enrichment analyses on 23 genes, which were primarily enriched in BP for the cellular polysaccharide biosynthetic process, polysaccharide biosynthetic process, and cellular carbohydrate biosynthetic process (Fig. 3A); CC is primarily enriched in the sarcomere, myofibril, and lateral plasma membrane; and MF is primarily enriched in tropomyosin binding, nuclear receptor activity (Fig. 3B).

### Screening for myopia-related biomarkers

**Fig. 4:**
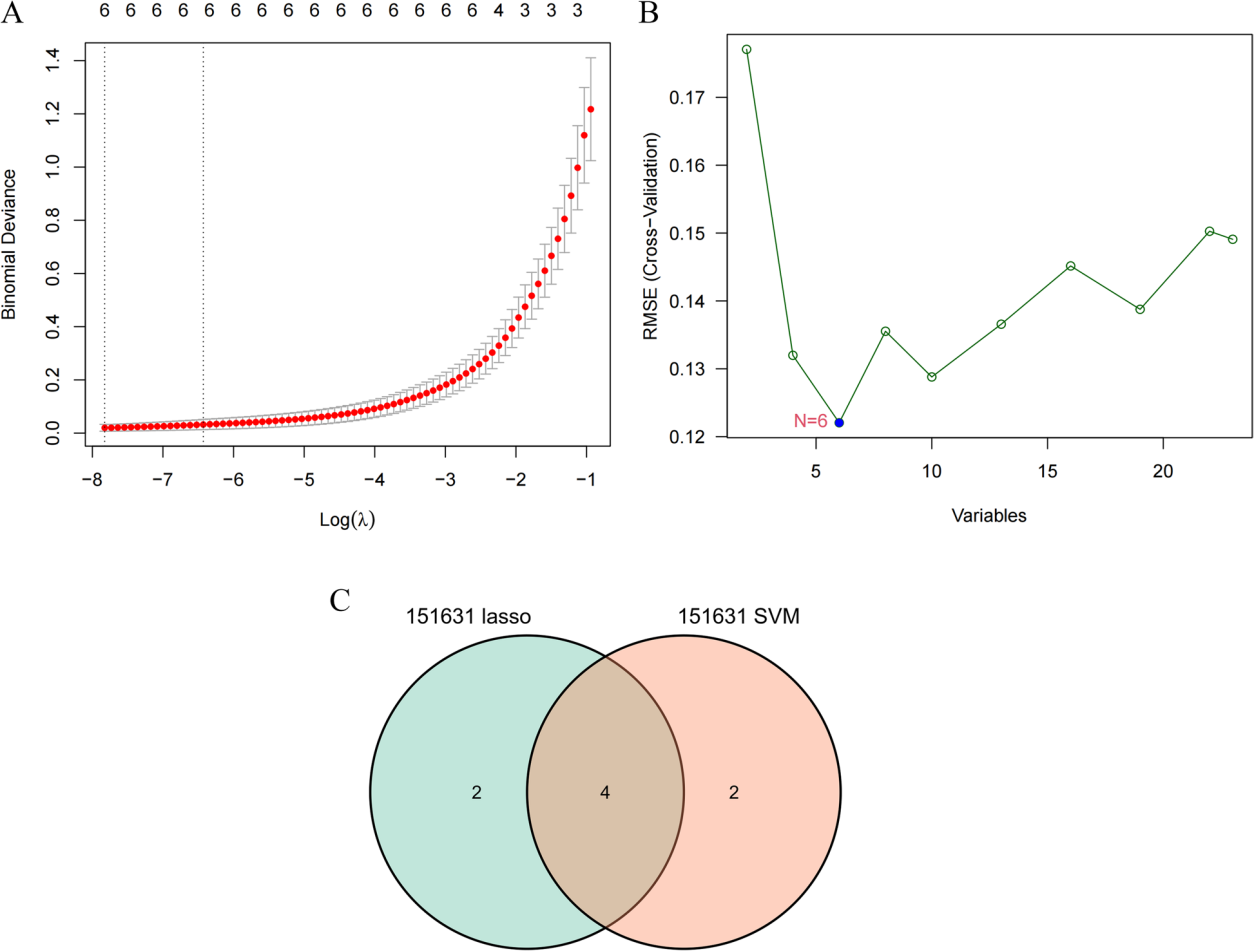
Myopia-related biomarker screening. **A** LASSO regression analysis. **B** SVM-RFE analysis. **C** Venn diagram of genes obtained from LASSO regression analysis and SVM-RFE analysis screening

We used two machine learning algorithms, LASSO and SVM-RFE, to screen myopia-related biomarkers in GSE151631: the LASSO regression algorithm selected six potential biomarkers (Fig. 4A) (NR1D1, PPP1R18, RTKN, LMOD1, PGBD2, and PPP1R3D); the SVM-RFE algorithm screened also 6 potential biomarkers (Fig. 4B) were obtained (PPP1R18, NUPR1, NR1D1, PPP1R3D, PGBD2, and ZNF780A). The biomarkers selected by both algorithms were plotted in a Venn diagram (Fig. 4C), and a total of 4 genes (NR1D1, PPP1R18, PGBD2, PPP1R3D) were identified as biomarkers of myopia in the overlapping part.

### Diagnostic efficiency of biomarkers

**Fig. 5:**
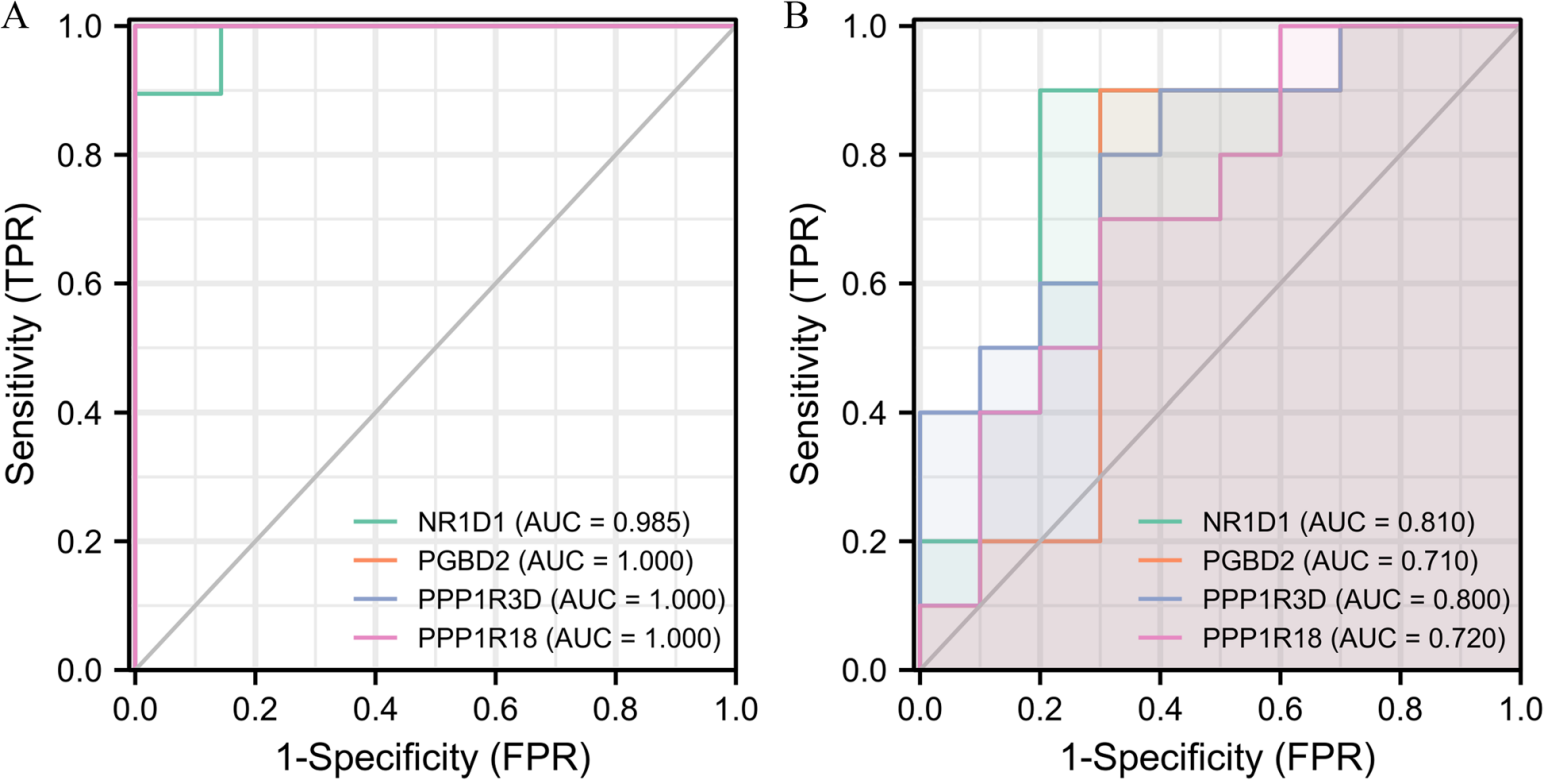
Diagnostic efficiency of myopia-related biomarkers. **A** Four biomarker ROC curves in GSE151631. **B** Four biomarker ROC curves in GSE112155

To clarify the diagnostic efficiency of the four genes in NR1D1, PPP1R18, PGBD2, and PPP1R3D, respectively, we subsequently plotted ROC curves and calculated the area under the curve (AUC) using GSE151631 and validated them in GSE112155. NR1D1 (AUC = 0.986) in GSE151631, PGBD2 (AUC = 1.000), PPP1R3D (AUC = 1.000), PPP1R18 (AUC = 1.000) (Fig. 5A); NR1D1 (AUC = 0.810), PGBD2 (AUC = 0.710), PPP1R3D (AUC = 0.800), in GSE112155 PPP1R18 (AUC = 0.720) (Fig. 5B). This result indicates that all four biomarkers have good diagnostic efficiency.

### Biomarker-related pathway prediction

**Fig. 6:**
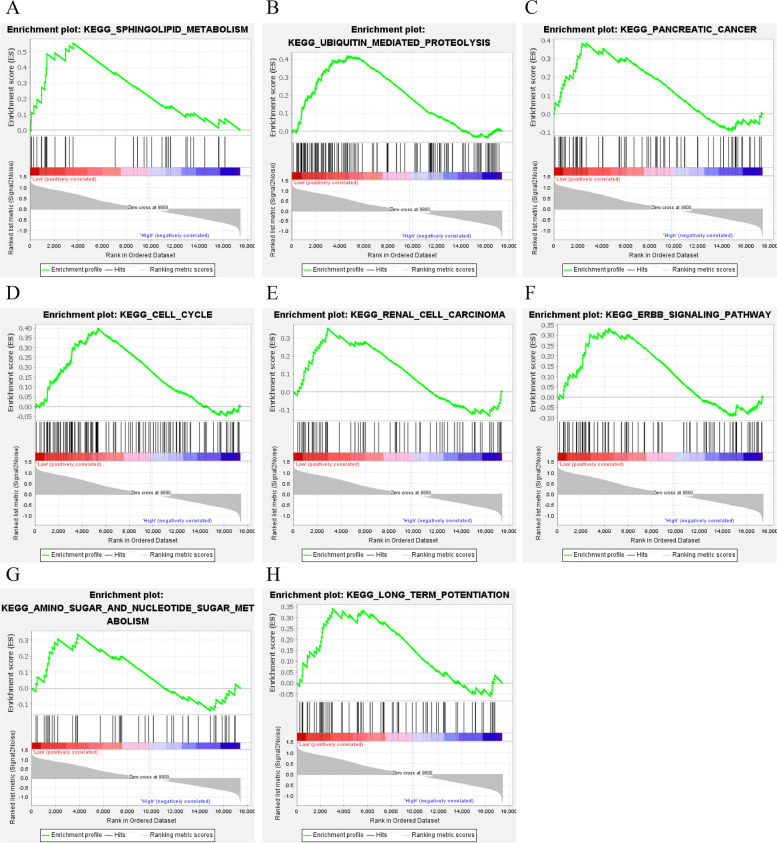
NR1D1 single gene GSEA

To predict potentially relevant pathways for the biomarkers, we combined GSE151631 and GSE112155 and performed GSEA based on the expression of each of the four biomarkers. eight pathways were enriched in the NR1D1 low expression group (Fig. 6): KEGG_SPHINGOLIPID_METABOLISM (NES = 1.89, *P* = 0.006), KEGG_UBIQUITIN_MEDIATED_PROTEOLYSIS (NES = 1.69, *P* = 0.000), KEGG_PANCREATIC_CANCER (NES = 1.64, *P* = 0.015), KEGG_CELL_CYCLE (NES = 1.57, *P* = 0.048), KEGG_RENAL_CELL_CARCINOMA (NES = 1.53, *P* = 0.039), KEGG_ERBB_SIGNALING_PATHWAY (NES = 1.47, *P* = 0.038), KEGG_AMINO_SUGAR_AND_NUCLEOTIDE_ SUGAR_METABOLISM (NES = 1.47, *P* = 0.030), and KEGG_LONG_TERM_POTENTIATION (NES = 1.43, *P* = 0.040). This result suggests that NR1D1 may play a negative regulatory role in these pathways.

**Fig. 7:**
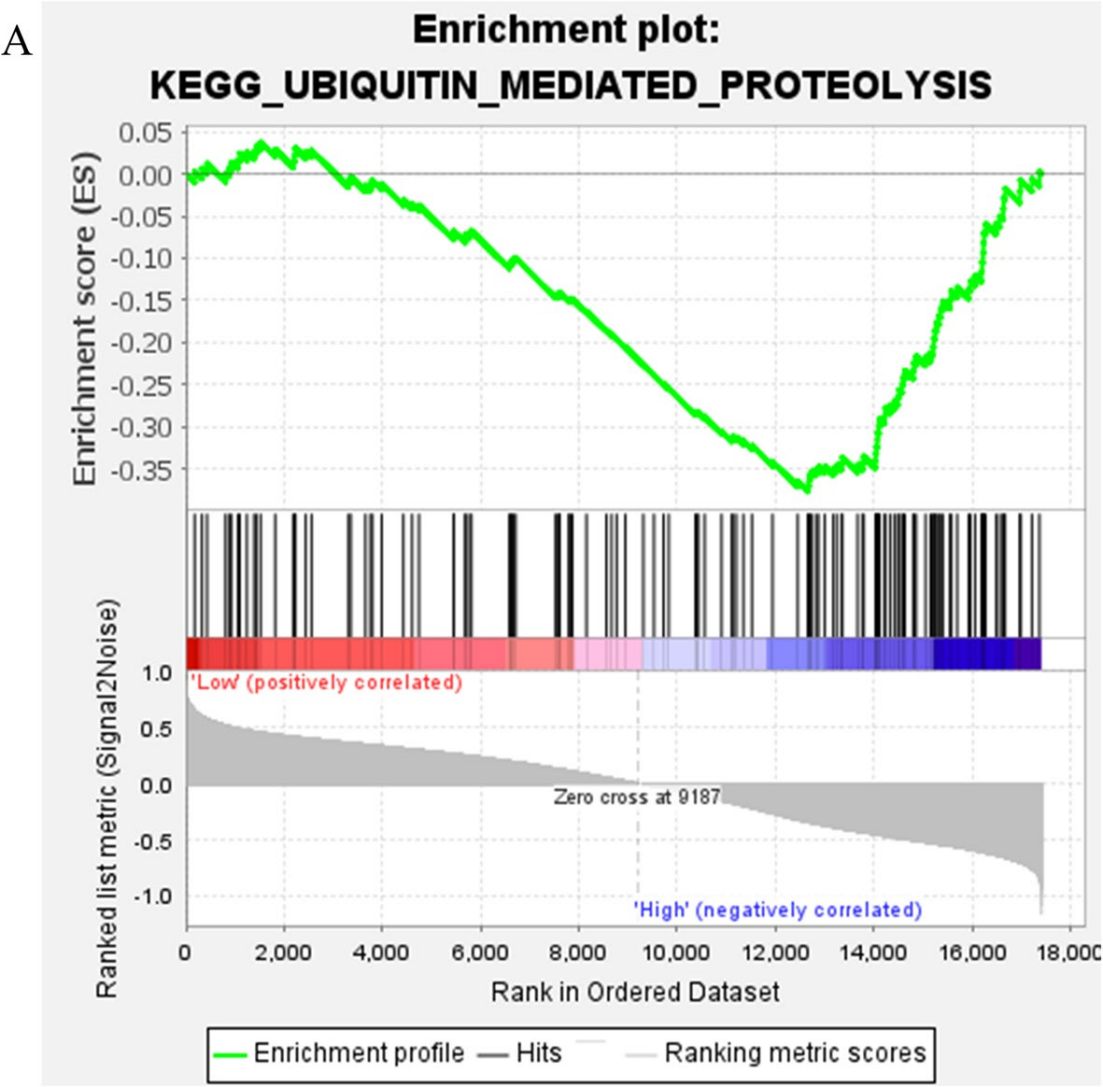
PGBD2 single gene GSEA

The PGBD2 high expression group was enriched to 1 pathway: KEGG_UBIQUITIN_MEDIATED_PROTEOLYSIS (NES=-1.52, *P* = 0.032) (Fig. 7). This suggests that PGBD2 may play an important role in UBIQUITIN_MEDIATED_PROTEOLYSIS.

**Fig. 8:**
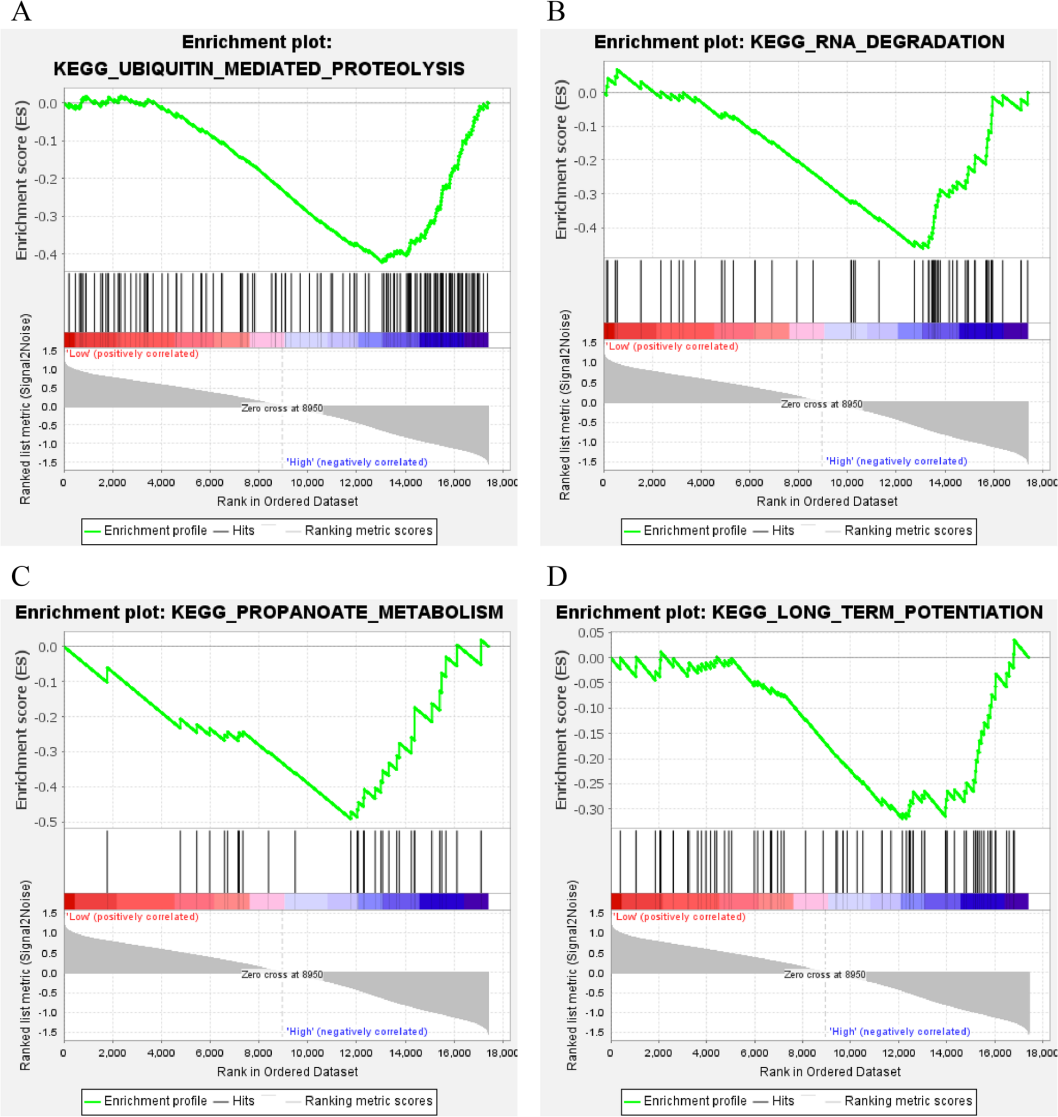
PPP1R3D single gene GSEA

The PPP1R3D high expression group was enriched to 4 pathways (Fig. 8): KEGG_UBIQUITIN_MEDIATED_PROTEOLYSIS (NES=-1.77, *P* = 0.000), KEGG_RNA_DEGRADATION (NES=-1.55, *P* = 0.018), KEGG_ PROPANOATE_METABOLISM (NES=-1.53, *P* = 0.047), and KEGG_LONG_TERM_POTENTIATION (NES=-1.35, *P* = 0.043). These results imply that PPP1R3D has a positive regulatory effect on these pathways.

**Fig. 9:**
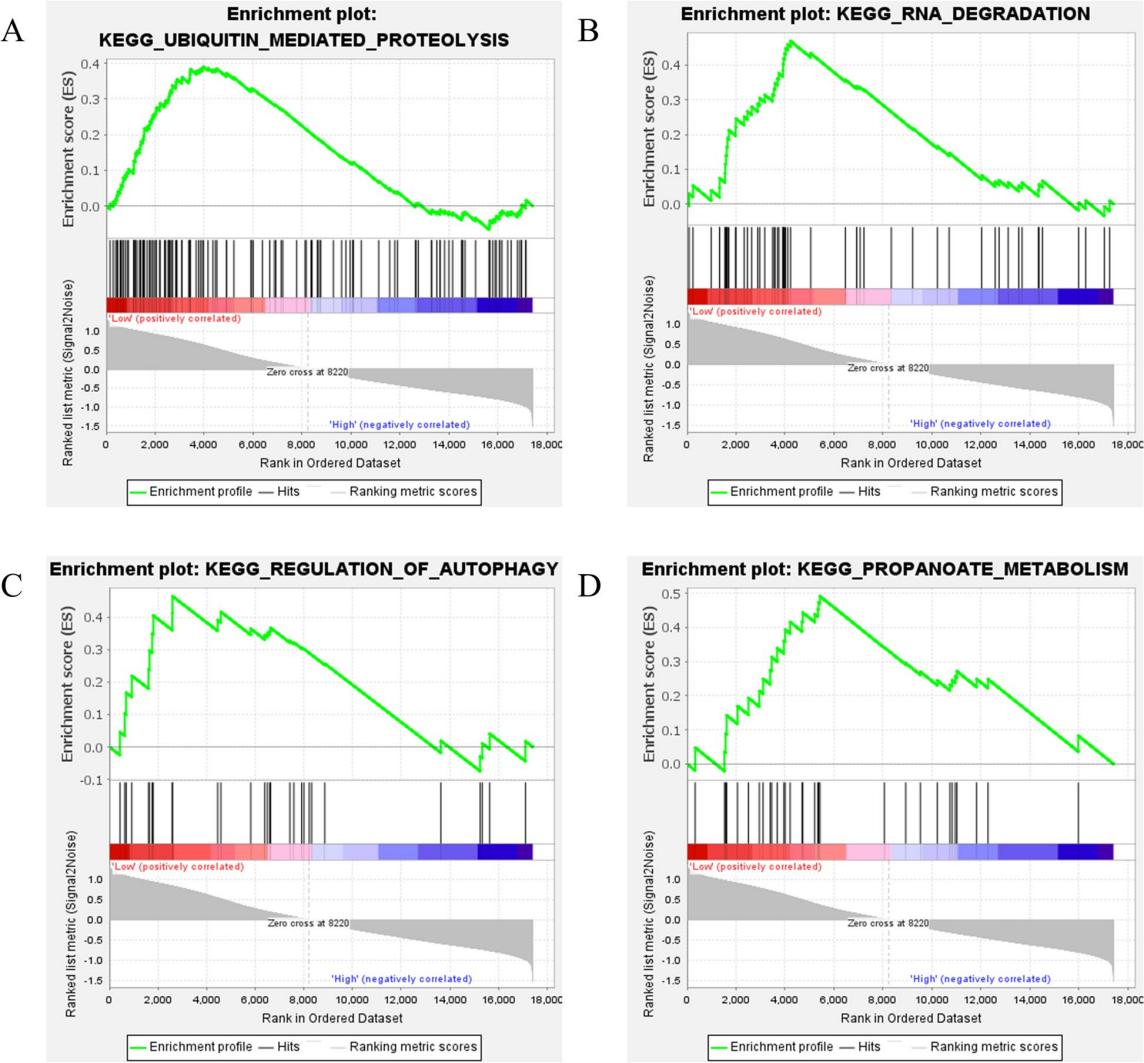
PPP1R18 single gene GSEA

The PPP1R18 low expression group was enriched to 4 pathways (Fig. 9): KEGG_UBIQUITIN_MEDIATED_PROTEOLYSIS (NES = 1.59, *P* = 0.015), KEGG_RNA_DEGRADATION (NES = 1.54, *P* = 0.025), KEGG_ REGULATION_OF_AUTOPHAGY (NES = 1.51, *P* = 0.049), and KEGG_PROPANOATE_METABOLISM (NES = 1.49, *P* = 0.031). These results suggest that PPP1R3D may inhibit the activation of these pathways.

## Discussion

There is growing evidence confirming that myopia is not simply a refractive error, but is influenced by many factors [13]. In this study, we compared the gene expression profiles of myopic patients’ corneas to those from normal populations. We searched both datasets for differentially expressed genes, and then we merged the results to uncover co-regulated genes. This analysis revealed 23 co-regulated genes to be myopia-related differentially expressed genes. The generation of carbohydrates is primarily impacted by the 23 distinct genes indicated above, which are involved in polysaccharide biosynthesis. High glucose levels may impact the glycosylation of corneal fibers and collagen cross-links in the corneal stroma, limiting the biomechanical weakening of the cornea and lowering the occurrence of conical corneas, according to earlier research [14, 15], while the body’s blood glucose levels can be influenced by the processes of carbohydrate synthesis and polysaccharide synthesis, which are enriched by separate genes, which is consistent with earlier research. Subsequent machine-learning analysis revealed four genes—NR1D1, PPP1R18, PGBD2, and PPP1R3D—as potential myopia biomarkers, all demonstrating robust diagnostic efficiency.The single gene GSEA results for the aforementioned four genes reveal that each of these four genes has an impact on the pathway for ubiquitin-mediated protein hydrolysis. All eukaryotic cells contain ubiquitin, which alters proteins for proteasomal breakdown and non-protein hydrolysis processes [16]. The ubiquitin protein hydrolysis system plays important role in the cell. These include regulation of the cell cycle, regulation of immune and inflammatory responses, control of signal transduction pathways, development, and differentiation [17]. These complex processes are controlled by the specific degradation of a protein or group of proteins. The role of ubiquitination in ophthalmology has been studied in several ways. In a study by Fu SH et al. [18], the epithelial-mesenchymal transition and cell permeability of retinal pigment epithelial cells were discovered to be impacted by the ubiquitination degradation process, which has an impact on diabetic retinopathy. In a study by Annika N Boehm et al. [19], it was discovered that in inflammatory eye diseases, the human leukocyte antigen (HLA)-F adjacent transcript 10 (FAT10) family of ubiquitin-like modifiers can lead to the loss of phosphodiesterase 6 (PDE6) by targeting PDE6 for proteasomal degradation through the formation of covalent covalent bonds. All of the myopia-related biomarkers examined in this investigation alter ubiquitin-mediated protein hydrolysis, but more research is required to determine the precise role of ubiquitin-mediated protein hydrolysis in the onset and progression of myopia.

NR1D1 is involved in metabolism, autophagy, cell proliferation, inflammation and other processes and regulates a variety of diseases [20–22]. It is not only a regulator of circadian clock metabolism, but also an important nuclear receptor for the normal function of mammalian retina [23]. Importantly, it can also regulate the expression of many genes in the retina [24, 25]. Studies have confirmed that NR1D1 reverses the functional NR2E3 gene in retinal degeneration mice. Therefore, NR1D1 can be used as a new therapeutic drug for retinal degeneration [23]. Additionally, it was shown that NR1D1 reduced retinal inflammation and prevented the activation of microglia linked to the start of retinal inflammation [26]. Protein phosphatase 1(PP1) is a major serine/threonine phosphatase that is expressed in all eukaryotic cells [27]. Previous research has revealed that the PP1-binding proteins protein phosphatase 1 regulatory subunit 18 (PPP1R18) and PPPIR subunit 3D (PPP1R3D) play a critical role in regulating vertebrate studies of development [28]. In addition, PP1 plays a key role in both the lens and human retinal epithelium [29]. PGBD2 is a member of the PiggyBac family [30], and there are few studies on the relationship between PGBD2 and myopia. The value of this gene in myopia diagnosis identified in this study may inspire subsequent studies.

In the current study, we compared patients with different degrees of myopia to normal cornea patients, searching for differentially expressed genes, investigating the functions of these genes, identifying key myopia biomarkers, studying the diagnostic efficacy of these key biomarkers, and based on GSEA analysis, identifying several key pathways that may be involved in myopia progression. These findings have contributed to our understanding of the pathophysiology of myopia. However, due to the limited sample size in this study, the strength of the evidence is reduced. We will use this research as a stepping stone for more clinical and basic experimental studies to further validate our findings, as the exact mechanisms of myopia are still largely unknown.

To further understand the potential roles of these genes in high myopia, future research should consider using larger sample populations and including more patients with high myopia. We will also explore whether these genes are associated with high myopia. Additionally, we plan to further investigate how these genes influence cellular functions and how they may interact with environmental factors to affect the severity of myopia. Through such efforts, we hope to gain a better understanding of the genetic basis of high myopia and potentially guide future treatment strategies.

## Conclusion

Our study shows that NR1D1, PPP1R18, PGBD2, and PPP1R3D are effective as biomarkers in the diagnosis of myopia and that NR1D1, PPP1R18, PGBD2, and PPP1R3D may be potential therapeutic targets.

### Supplementary Information

**Additional file 1.**

## Data Availability

The available data has been placed in the Supplementary materials. Web links and URLs: The GEO database (https://www.ncbi.nlm.nih.gov/geo/). The GSEA database (http://www.gsea-msigdb.org/gsea/index.jsp).

## Abbreviations

AUC: Area Under Curve
GSA: Gene Set Analysis
GWAS: Genome-wide association study
ORA: Over representation analysis
FDM: Form-deprivation myopia
NGS: Next-generation sequencing
GEO: Gene Expression Omnibus
TPM: Transcripts per kilobase million
GO: Gene Ontology
KEGG: Kyoto Encyclopedia of Genes and Genomes
CC: Cell composition
MF: Molecular function
SVM-RFE: Support Vector Machine Recursive Feature Elimination
LASSO: Least Absolute Shrinkage and Selection Operator
ROC: Receiver operating characteristic
HLA: Human leukocyte antigen
FAT: F adjacent transcript
PDE: Phosphodiesterase
PPI: Protein phosphatase 1
PPP1R: Protein phosphatase 1 regulatory
